# Improving Tableting Performance of Lactose Monohydrate by Fluid-Bed Melt Granulation Co-Processing

**DOI:** 10.3390/pharmaceutics13122165

**Published:** 2021-12-15

**Authors:** Djordje Medarević, Jelena Djuriš, Mirjana Krkobabić, Svetlana Ibrić

**Affiliations:** Department of Pharmaceutical Technology and Cosmetology, Faculty of Pharmacy, University of Belgrade, Vojvode Stepe 450, 11221 Belgrade, Serbia; jelena.djuris@pharmacy.bg.ac.rs (J.D.); mirjana.krkobabic@pharmacy.bg.ac.rs (M.K.); svetlana.ibric@pharmacy.bg.ac.rs (S.I.)

**Keywords:** co-processing, direct compression, lactose monohydrate, fluid-bed melt granulation, self-lubricating excipients, tableting behaviour

## Abstract

Co-processing is commonly used approach to improve functional characteristics of pharmaceutical excipients to become suitable for tablet production by direct compression. This study aimed to improve tableting characteristics of lactose monohydrate (LMH) by co-processing by fluid-bed melt granulation with addition of hydrophilic (PEG 4000 and poloxamer 188) and lipophilic (glyceryl palmitostearate) meltable binders. In addition to binding purpose, hydrophilic and lipophilic excipients were added to achieve self-lubricating properties of mixture. Co-processed mixtures exhibit superior flow properties compared to pure LMH and comparable or better flowability relative to commercial excipient Ludipress^®^. Compaction of mixtures co-processed with 20% PEG 4000 and 20% poloxamer 188 resulted in tablets with acceptable tensile strength (>2 MPa) and good lubricating properties (ejection and detachment stress values below 5 MPa) in a wide range of compression pressures. While the best lubricating properties were observed when glyceryl palmitostearate was used as meltable binder, obtained tablets failed to fulfil required mechanical characteristics. Although addition of meltable binder improves interparticle bonding, disintegration time was not prolonged compared to commercial excipient Ludipress^®^. Co-processed mixtures containing 20% of either PEG 4000 or poloxamer 188 showed superior tabletability and lubricant properties relative to LMH and Ludipress^®^ and can be good candidates for tablet production by direct compression.

## 1. Introduction

Due to their numerous advantages, such as high physical and chemical stability, the precision of dosage, simple administration, portability, cheap large-scale production, and suitability to provide different release patterns of active ingredients, tablets are still the most common pharmaceutical dosage form on the market [1,2]. Tablets can be produced by several different methods, but direct compression is a method of the first choice, owing to its simplicity, requiring only mixing of ingredients prior to compression in tablets. Although it is the most desired method for tablets production, direct compression requires good flow and compression characteristics of the mixture prepared for tableting [3,4]. It has been estimated that only about 20% of tableting mixtures pose required characteristics to be directly compressed in tablets [2]. For mixtures that do not fulfil necessary prerequisites for processing by direct compression, wet or dry granulation is applied before compression, in order to improve powder flow and/or compression characteristics. However, inclusion of additional steps in the tablet production process increases the overall cost of the process and extends its duration. Exposure of powder mixture to water and increased temperature during the wet granulation process can also lead to degradation of susceptible components. Since most active pharmaceutical ingredients are low-dosed, flow and compression characteristics of tableting mixture are mostly affected by diluents which comprise the major part of the mixture [5]. Different approaches have been applied in order to improve flow and compression properties of diluents to make them suitable for using in direct compression. The development of completely novel excipients is seldom used as an approach for improving tableting performances, due to huge costs, long duration, and requirements for toxicological studies. Physical properties of existing excipients can be also modified by granulation, grinding, spray drying, and similar techniques in order to make excipients suitable for direct compression. Although modification of physical properties is simple, the extent of improvement of tableting performances is limited and it is possible that improvement of one characteristic will lead to impairment of other characteristics important for tableting [5,6]. Co-processing implies the combination of two or more established excipients in some common process (granulation, spray drying, milling, co-crystallization, etc.) in order to synergistically improve excipient functional properties and mask undesired properties. This concept is based on component interactions on the subparticle level, wherein particles of one excipient can be incorporated on the surface or within the core of particles of another excipient. Since no chemical changes occur during co-processing, the obtained product can be considered as a mixture of the existing excipient and thus is not subjected to extensive toxicological studies [6].

Due to its low cost and huge availability from the dairy industry, physical and chemical stability, water solubility, low hygroscopicity, and bland taste, lactose is still one of the most commonly used tablet diluents. However, poor flow and binding properties make lactose monohydrate unsuitable for application in tablet formulations which are produced by direct compression [2]. Different grades of lactose monohydrate with improved flowability and compactibility have been produced by granulation and spray drying [7]. Although these grades are intended for tablet production by direct compression, they still exhibit some drawbacks which cannot be eliminated by changing the physical properties of the initial lactose monohydrate. Formation of spherical particles and partial conversion from crystalline to amorphous form during spray drying contribute to improved flow and compaction properties of spray-dried lactose. However, the tensile strength of tablets produced by compressing of spray-dried lactose is often not sufficient, while poor dilution capacity limits its application to formulation with low-dose drugs. Additionally, the content of amorphous lactose, which is responsible for binding properties due to plastic deformation under pressure, can significantly vary depending on the spray drying process, leading to variation in tablet properties [8].

Limitations of physically modified grades of lactose monohydrate moved the focus of the excipient industry towards the development of co-processed excipients. Numerous co-processed excipients based on lactose monohydrate have been developed wherein lactose is combined with plastically deforming diluents (microcrystalline cellulose, powder cellulose, etc.), binders (povidone, hypromellose, etc.), and disintegrants (crospovidone, starch, etc.) [4]. However, most of the co-processed excipients containing lactose monohydrate require addition of external lubricants to ensure tablet removal from the die without damage. Since lubricant action of most commonly used lubricants is based on the formation of hydrophobic film around particles, biopharmaceutical and mechanical properties of produced tablets can be seriously compromised [9]. Therefore, the design of the excipients with lubricant properties (i.e., self-lubricating excipients) is one of the imperatives in the development of novel co-processed excipients. Such excipient is Lubritose^®^ SD, containing spray-dried lactose as a major component and glyceryl monostearate which acts as a lubricant [10]. Madhvi et al. described preparation of the co-processed excipient containing lactose monohydrate, mannitol, crospovidone, and polyethylene glycol 4000 by melt granulation [11]. Similarly, Gohel and Jogani described the development of a co-processed excipient for direct compression by melt granulation of a mixture of lactose monohydrate and microcrystalline cellulose with a binder mixture containing polyethylene glycol 4000 and povidone K30 [12]. However, both of the previous studies were performed using simple manual mixing of the components, without considering industrially feasible techniques, and also there is no information provided regarding the quantitative assessment of lubricating properties. According to the authors’ knowledge, there are no studies which comparatively investigate development of self-lubricating excipients suitable for direct compression, with lactose monohydrate as the major component and hydrophilic or lipophilic meltable binders by fluid-bed melt granulation. The major advantage of co-processing based on the fluid-bed melt granulation is suitability for implementation in the majority of pharmaceutical industry, due to usage of equipment that is commonly available, without substantial modifications. Additional benefits of co-processing by melt granulation include short processing time, high product yield, and absence of using of solvents [11]. Therefore, this study aims to develop a self-lubricating co-processed excipient for direct compression by fluid-bed melt granulation using either hydrophilic or lipophilic meltable binders which also act as lubricants. Additionally, tableting properties of co-processed mixtures were assessed by a dynamic powder compaction analyser, with particular emphasis on the evaluation of lubricant properties.

## 2. Materials and Methods

### 2.1. Materials

α-lactose monohydrate (LMH, Sigma Aldrich, St. Louis, MO, USA), polyethylene glycol 4000 (PEG 4000, Sigma Aldrich, St. Louis, MO, USA), poloxamer 188 (P188, Kolliphor^®^ P188, BASF, Ludwigshafen, Germany), glyceryl palmitostearate (GPS, Precirol^®^ ATO 5, Gattefossé, Saint-Priest, France), croscarmellose sodium (Primellose^®^, DFE Pharma, Goch, Germany), calcium dihydrogen phosphate dihydrate (Emcompress^®^, JRS Pharma, Rosenberg, Germany) have been used for the preparation of co-processed excipients. Commercially available co-processed excipient Ludipress^®^ (BASF, Ludwigshafen, Germany) has been used for comparison with the prepared co-processed mixtures.

### 2.2. Methods

#### 2.2.1. Co-Processing by Fluid-Bed Melt Granulation

Co-processing of mixtures was performed by in situ fluid-bed melt granulation using Mycrolab fluid-bed system (OYSTAR Hüttlin, Schopfheim, Germany). 220 g of mixtures (Table 1) was granulated with the following setting of process parameters: Inlet air temperature and inlet airflow rate were set at 70 °C and 20 m^3^/h, respectively, while the granulation process lasted for 10 min after product temperature reached 60 °C. PEG 4000, P188, and GPS were sieved before granulation, and fractions with particle size between 180 and 350 μm were used in the granulation process.

#### 2.2.2. Particle Size Distribution Determination

Particle size distribution of co-processed mixtures was determined by sieves analysis, using the vibratory sieve shaker (Erweka, Heusenstamm, Germany) and a set of standard sieves in the range of 180–1250 μm. Analysis was performed on a sample mass of 100 g for 10 min.

#### 2.2.3. Flow through an Orifice

Flowability of co-processed mixtures was determined according to the procedure described in the European Pharmacopoeia 10.0 chapter 2.9.51 [13]. Sample mass of 30 g was transferred to the flow testing device (Flow meter GDT, Erweka, Heusenstamm, Germany) and the time necessary for sample to flow through 12 mm orifice was recorded. Flowability was expressed as the sample flow rate, calculated as a ratio of sample mass and time for sample flow through an orifice. All measurements were performed in triplicate and the results were expressed as mean ± standard deviation.

#### 2.2.4. Determination of Bulk and Tapped Density, Compressibility Index and Hausner Ratio

Determination of bulk and tapped density was performed according to chapter 2.9.34. of European Pharmacopoeia 10.0 [13]. Sample mass of 20 g was transferred to 50 mL graduated cylinder and bulk density was calculated as the ratio of sample mass and volume occupied by the sample. Tapped density was determined as the ratio of sample mass and volume read after 1250 taps in a Stampfvolumeter STAV2003 instrument (J. Engelsmann AG, Ludwigshafen, Germany).

Compressibility index (CI) and Hausner ratio were calculated according to Equations (1) and (2), respectively:

CI (%) = 100 × (tapped density-bulk density)/tapped density(1)

Hausner ratio = tapped density/bulk density(2)

All measurements were performed in triplicate and the results were expressed as mean ± standard deviation.

#### 2.2.5. Evaluation of Tableting Behaviour

A benchtop single-punch dynamic powder compaction analyser Gamlen D500 (Gamlen Instruments, Biocity Nottingham, UK) was used to study sample compaction behaviour. It has been previously demonstrated that Gamlen D500 can be used as a useful tool to simulate tablet compression process on the large-scale rotary tablet press [14]. In the first stage, samples (100 mg) were manually filled in the die and compressed by flat-faced 6 mm punch at punch speed of 60 mm/min. Compression was performed within the range of loads from 100 to 500 kg, which corresponds to compression pressures from 34.7 to 173.5 MPa. In the second stage, the tablet was detached from the die base. In the ejection phase, the tablet was pushed through the bottom of the die in the tablet holder and removed from the instrument. The instrument was fully operated by the software which generates load vs. punch position curves during all three phases. Work of the compression (WC), detachment (DW), and ejection (EW) phases were calculated as the area under the force vs. displacement curve in each phase. Net work of compression (NWC) was calculated by subtracting work of elastic recovery from the WC. In die elastic recovery (IER) was used as an indicator of material elastic properties and calculated according to the following Equation (3):

IER (%) = 100 × (*t_max_* − *t_min_*)/*t_min_* (3)

where *t_max_* is tablet thickness at the end of compression phase, calculated as the difference between base punch position and punch displacement corresponding to zero compression load at the end of the compression;

*t_min_* is tablet thickness corresponding to maximum punch displacement during compression, calculated as the difference between base punch position and maximum punch displacement during compression.

Lubricant properties of materials were assessed from the parameters ejection work (EW), as described above and ejection stress (ES), calculated according to the following Equation (4) [15]:

ES = *F_e_*/π·*D·h* (4)

where *F_e_* is maximum force recorded during tablet ejection, *D* is tablet diameter and *h* is tablet thickness.

The tendency of tablets to stick to die base was estimated using detachment work (DW), calculated from the force vs. displacement curve in the detachment phase, and detachment stress calculated using Equation (5):

DS = *F_d_*/(*D*/2)^2^·π (5)

where *F_d_* is maximum force recorded during tablet detachment.

Tablet diameter (*D*), thickness (*h*), and breaking force (*F*) were determined immediately after tablet preparation. Erweka TBH 125D tablet hardness tester (Erweka, Heusenstamm, Germany) was used for the determination of tablet breaking force and diameter, while the thickness of the tablets was determined by the caliper. Obtained parameters were used for the calculation of tablet tensile strength, according to the following Equation (6) [16]:

σ = 2*F*/π·*D·h* (6)

All parameters used for evaluation of tableting behaviour were expressed as mean ± standard deviation of six replicates. Tensile strength above 2 MPa is considered as sufficient for tablets to withstand mechanical stress during production and handling [14]. Ejection and detachment parameters of co-processed mixtures and LMH and Ludipress^®^ were compared by means of Student’s t-test for normally distributed data or Mann-Whitney U test for data that are not normally distributed to assess if there are statistically significant differences (*p* < 0.05). Statistical analysis was performed using SPSS 20.0 package (SPSS Inc., Chicago, IL, USA).

#### 2.2.6. Tablet Disintegration

Tablet disintegration testing was performed using Erweka ZT 52 disintegration tester (Erweka, Heusenstamm, Germany), according to the procedure described in European Pharmacopoeia 10.0 for uncoated tablets [13]. Six tablets prepared by compression of LMH, Ludipress^®^, or each of the co-processed mixtures at a compression pressure of 173.5 MPa were tested in purified water at 37 ± 1 °C. The time taken for the last tablet to disintegrate was recorded as a disintegration time.

## 3. Results and Discussion

### 3.1. Particle Size Determination

**Figure 1:**
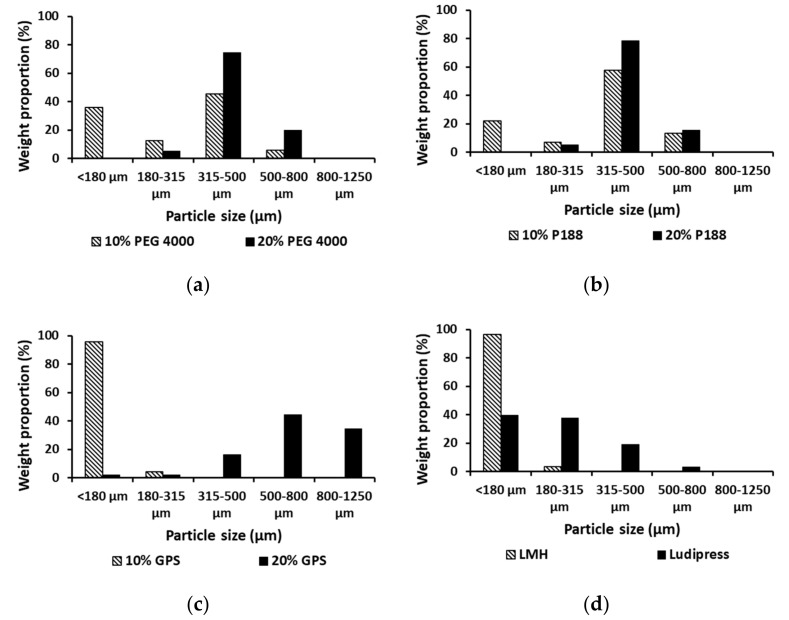
(**a**–**d**) Particle size distribution of co-processed mixtures, LMH and Ludipress^®^.

Particle size analysis showed that melt granulation resulted in significant size enlargement for most of the granulated mixtures (Figure 1). Fraction of particles with a size below 180 μm was dominant only in the case of the mixture containing 10% of GPS which is similar to raw LMH. Increase in the proportion of meltable binder led to increase of the particle size of granulated mixtures. It can be observed that granulation with 20% of P188 and PEG 4000 gave a product with very uniform particle size and the dominant fraction of particles with a size between 315 and 500 μm. On the other hand, a granulated mixture containing either 10% of P188 or PEG 4000 contained a considerable amount of particles which size is below 180 μm. While particles with size below 180 μm were dominant for sample processed with 10% of GPS, using of 20% of GPS resulted in excessive agglomeration.

### 3.2. Flowability Testing

Good flow properties are essential for excipients used for tablet preparation by direct compression. Flowability testing (Table 2) showed very poor flow properties of raw LMH, which precludes its use as a diluent for direct compression. Although the flowability of commercial co-processed excipient Ludipress^®^ was significantly better, more pronounced improved flow properties were observed for mixtures co-processed by melt granulation. Only mixture with 10% of GPS showed very similar flow properties to Ludipress^®^, which is probably a consequence of the significant amount of fine particles in this mixture. Although an increase in particle size should result in a higher flow rate, slightly higher flow rates were determined for samples with 10% of PEG 4000 and P188, which contains a higher amount of fine particles, relative to samples with 20% of PEG 4000 and P188. However, all samples granulated with either 10 or 20% of PEG 4000 and P188 showed very favourable flow properties, which are several orders of magnitude higher than raw LMH.

### 3.3. Evaluation of Tableting Behaviour

**Figure 2:**
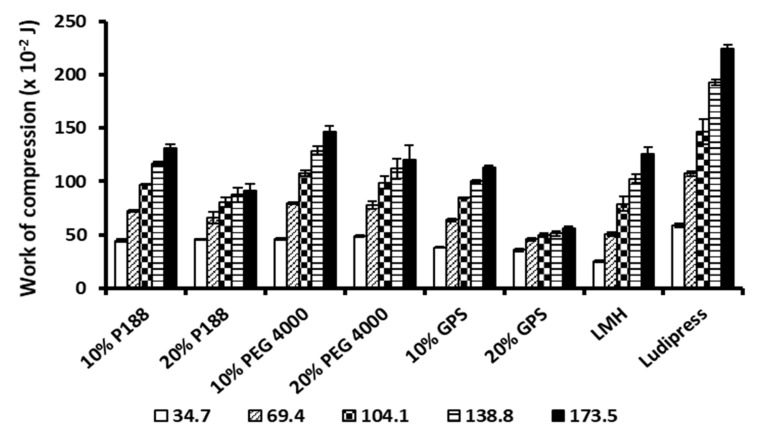
Calculated net work of compression for co-processed mixtures, LMH and Ludipress^®^ in the range of compression pressures 34.7–173.5 MPa.

Calculated NWC values for co-processed mixtures, LMH and Ludipress^®^ are shown in Figure 2. It is clearly evident that NWC values increase with a rise in applied compression pressure, as expected due to higher energy input at higher pressures. Presented results show considerable higher NWC for Ludipress^®^ within the studied range of compression pressures, relative to all other samples. An increase in the proportion of meltable binder led to less increase in NWC with a rise in compression pressure. This is particularly pronounced for the sample with 20% of GPS, where only slight differences of NWC were observed at different compression pressures. Higher NWC values of samples containing less binder can be attributed to a lower particle size of these samples relative to those containing a higher amount of binder and consequently higher surface area of contact between particles, which increase resistance to deformation. This explains the lower NWC for 20% GPS sample, as it contains more than 80% (*w/w*) particles with a size above 500 μm. Although high NWC is often attributed to materials that undergo high extent of deformation under compression pressure and produce very strong tablets, it can also result from high friction between compressed material and metal part of tableting equipment. Despite favourable deformation properties of some materials, their application for tablet production can be seriously compromised due to high friction with the part of the equipment or high elastic recovery upon the termination of compression. Therefore, all materials were also analysed in terms of elastic recovery, tensile strength, ejection, and detachment parameters.

**Figure 3:**
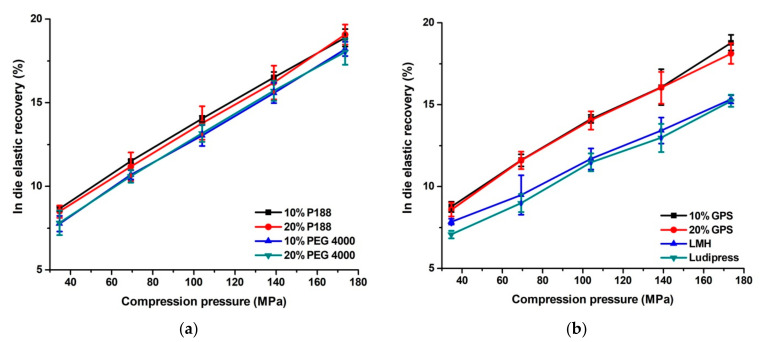
(**a**,**b**) In die elastic recovery for co-processed mixtures, LMH and Ludipress^®^.

IER linearly increases for all tested materials (Figure 3) with a rise in compression pressure, as higher compression pressures cause that materials store more elastic energy which is released in the decompression stage. LMH and Ludipress^®^ showed very similar IER, which are slightly lower compared to all co-processed mixtures. Since both LMH and Ludipress^®^ are mainly deformed by brittle fracture [2,17], problems with a high extent of elastic relaxation are not expected. IER values for all co-processed mixtures were in the range between 7.8 and 19.1% and closely match each other at the corresponding compression pressures. A high extent of elastic relaxation is undesirable for excipients use for tablet preparation, as it leads to the weakening of interparticle bonds which can further cause a decrease of tablet mechanical strength or in the worst case tablet capping or lamination. Therefore, IER can be a useful parameter, but only in conjunction with tablet mechanical characterization.

**Figure 4:**
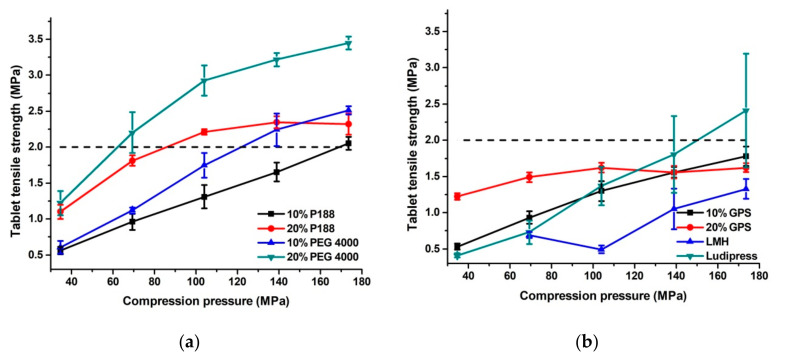
(**a**,**b**) Tabletability profiles of co-processed mixtures, LMH and Ludipress^®^.

Considerable differences in tensile strength can be observed on tabletability profiles of tested mixtures (Figure 4). The lowest tensile strength was determined for tablets of pure LMH, as expected due to the well-known poor tableting properties of LMH. At the lowest compression pressure used in the study (69.4 MPa) obtained LMH tablets were characterized by very low mechanical resistance, which was not sufficient to withstand mechanical testing. Although compression of Ludipress^®^ resulted in tablets of higher tensile strength, relative to LMH, tablets with a tensile strength above 2 MPa were obtained only at the highest compression pressure applied in the study. Additionally, high deviations in tensile strength of replicated samples were observed for tablets made of Ludipress^®^. Tabletability profiles showed that it is possible to obtain tablets of tensile strength above 2 MPa by compression of samples granulated with PEG 4000 or P188. However, marked differences in tensile strength were observed depending on the type and proportion of the meltable binder. The trend of rising in tablet tensile strength with the increasing proportion of meltable binder is noticeable, particularly for mixtures containing PEG 4000, as a higher amount of binder increases interparticle bonding. An increase in tablet tensile strength with an increase in PEG 4000 concentration in the co-processed mixture has been also previously reported by Madhvi et al. [11]. The good tabletability of granulates produced by fluid-bed granulation is also often attributed to their high porosity and easy breakage into smaller particles during compression, which increase available bonding area [18,19]. The highest tensile strength within the whole range of compression pressures was observed for tablets prepared from a mixture with 20% PEG 4000. The particular advantage of this mixture is that acceptable tensile strength (>2 MPa) was achieved even when lower compression pressures (<70 MPa) were used. Although mixture with 20% P188 showed lower tensile strength, it reached target values of tensile strength at a compression pressure of ~104 MPa, and a further rise in compression pressure did not significantly affect tensile strength. Co-processed mixtures with 10% of PEG 4000 and P188 showed a rise in tensile strength with an increase in compression pressure, but target tensile strength was achieved at significantly higher compression pressures relative to mixtures with a higher amount of binder. The lowest tensile strength was achieved for samples granulated with lipophilic GPS and tablets prepared from these samples did not fulfil the set criteria for tensile strength. It is of particular importance that compacts prepared from mixtures with 20% of PEG 4000 and 20% P188 exhibit higher tensile strength compared with those prepared from mixtures with the lower amount of these binders, LMH or Ludipress^®^, despite lower work utilized for their compaction. This demonstrated better utilization of energy in the compression process of mixtures co-processed with 20% of PEG 4000 or P188.

Ejection and detachment parameters were calculated to evaluate sticking tendency between tablets and part of equipment and therefore to assess whether co-processed mixtures possess self-lubricating properties or the addition of external lubricants is necessary. Although there are no general requirements for ES and DS, values below 5 MPa are usually considered as acceptable in the design of the tableting process [15,20]. High ES and DS indicate on excessive sticking and friction between tablet and tableting tools, which can cause different kind of defects in prepared tablets, or even complete failure of tablets. All co-processed mixtures showed acceptable values of DS in the whole range of applied compression pressures (Table 3). The lowest DS was calculated for mixtures containing GPS. It is also evident that mixtures co-processed with PEG 4000 exhibit higher DS compared with mixtures where P188 was used as a meltable binder. Using of PEG 4000 as a meltable binder and lubricant failed to significantly improve DS values, with respect to both LMH and Ludipress^®^. Both LMH and Ludipress^®^ showed acceptable DS in the range of compression pressures 34.7–104.1 MPa, above which DS increases above the acceptable limit. Detachment work (DW) is also a useful parameter, as it considers energy applied in the whole detachment process and not only maximum detachment force, like DS. A similar trend in DW can be observed as for DS, but the values differences between co-processed mixtures and Ludipress^®^ or LMH were even higher. Significantly lower (*p* < 0.05) DW values relative to LMH and Ludipress^®^ were calculated for all co-processed mixtures in the range of compression pressures 104.1–173.5 MPa.

In the ejection step, force is applied on the tablet to eject it out of the die by overcoming residual die wall stress and friction between the tablet and die wall. Statistically significant lower ES and EW values (*p* < 0.05) were obtained for all co-processed mixtures with respect to both LMH in Ludipress^®^ in the whole range of applied compression pressures.

Obtained ES values increase with the rise in compression pressure for all samples (Table 4), as a higher amount of energy is stored in the compact and released upon the termination of compression pressure. Relaxation of compact in radial direction leads to higher residual die wall stress and thus higher ES [21]. Particularly high ES values were determined for LMH and Ludipress^®^. Despite tablets of acceptable tensile strength were obtained by compression of Ludipress^®^ at a compression pressure of 173.5 MPa, both high ES and DS preclude it from being using as direct compression diluent without the addition of lubricant. The same rank order in reducing ES was observed amongst three evaluated meltable binders, as in the case of DS. Mixtures containing GPS showed superior lubricant properties with ES < 1 MPa. The lowest ES was observed for mixture co-processed with 20% GPS, where ES was below 0.3 MPa. This further supports good lubricating properties of GPS. This sample was also characterized with the lowest NWC, so the lowest amount of energy is stored in the compact and thus released upon the termination of compression pressure. Great lubricating properties of mixtures co-processed by melt granulation with GPS have been also recently reported by Djuris et al. [22]. Despite the best lubricating properties, this sample did not fulfil the criteria for tensile strength, so it cannot be considered suitable for direct compression. Higher ES values were obtained for compacts prepared from mixtures containing PEG 4000 or P188, but they were still in the acceptable range below 5 MPa. Similarly like in the case of DS, P188 was more efficient in reducing ES compared to PEG 4000, while there was no clear dependence of ES from the concentration of either PEG 4000 or P188. The same rank order of EW values was observed as for the ES, but the differences between LMH and Ludipress^®^ on the one hand and co-processed mixtures on the other were several orders of magnitude higher relative to ES values. While ES values at a compression pressure of 173.5 MPa were 3–8 times lower for mixtures co-processed with P188 or PEG 4000 compared to Ludipress^®^, corresponding EW values were 18–41 times lower for these co-processed mixtures.

Compaction analysis of samples by Gamlen D500 dynamic powder compaction analyser showed that neither LMH nor Ludipress^®^ are suitable for tablet production by direct compression without the addition of lubricants. Granulation with each of three studied meltable binders produced mixtures with good self-lubricating properties, which can be compressed into tablets without the addition of external lubricants. Higher tablet tensile strength, together with acceptable ejection and detachment parameters make mixtures co-processed with 20% PEG 4000 or P188 as the most suitable for direct compression.

### 3.4. Tablet Disintegration Testing

The addition of meltable binder increases interparticle bonding which contributes to higher tablet tensile strength. Higher mechanical strength is a desirable tablet attribute for the prevention of tablet damage during the production process. However, higher interparticle bonding can prolong the disintegration of tablets and further delay drug release. Thus, disintegration testing was performed to investigate whether tablets prepared by compression of co-processed mixtures at the highest compression pressure used in this study fulfil requirements for the disintegration of immediate-release tablets. The obtained results (Table 5) showed the fastest disintegration of compacts made of LMH and co-processed mixture with 10% GPS due to lower interparticle bonding, which is manifested in low tensile strength. The longest disintegration time was measured for tablets prepared by compression of mixture with 20% of GPS, despite similar tensile strength of these tablets compared to those prepared from mixture containing 10% of GPS. However, a higher proportion of lipophilic GPS hinders water penetration within the tablet structure and thus prolongs tablet disintegration. Disintegration testing showed that the disintegration time of compacts prepared from mixtures co-processed with P188 and PEG 4000 was not significantly longer compared to commercial co-processed excipients Ludipress^®^. Disintegration time of tablets prepared from mixture with 20% of P188 was only slightly longer compared with those made of Ludipress^®^, while compacts prepared from other mixtures with P188 or PEG 4000 disintegrates even faster. Increase in the proportion of PEG 4000 and P188 prolonged tablet disintegration time, since higher amount of binder increases interparticle binding. In the end, it should be noted that disintegration time of tablets prepared from all mixtures containing PEG 4000 or P188 was far below the requirements of European Pharmacopoeia for the disintegration time of immediate release-tablets [13].

## 4. Conclusions

In this study, co-processed excipients suitable for direct compression which contain LMH as the main component and exhibit self-lubricating properties were successfully developed by fluid-bed melt granulation. Granulates obtained by co-processing with PEG 4000, P188, or GPS showed superior lubricating properties in the tablet compaction process, compared with pure LMH and commercial co-processed excipient Ludipress^®^. Amongst tested meltable binders, GPS showed the best lubricating properties, but compacts prepared from mixtures containing GPS did not satisfy requirements for mechanical characteristics. Mixtures granulated with 20% of PEG 4000 or P188 showed better tabletability and superior lubricating properties relative to Ludipress^®^, which altogether show their suitability for application in tablet production by direct compression. It is of particular importance that these co-processed mixtures were prepared by in situ fluid-bed melt granulation, which can be applied using equipment that is currently widely available in the pharmaceutical industry, without need for any modification. This makes the described process as cost-effective for wide application in the pharmaceutical industry.

## Figures and Tables

**Table 1:** Composition of the mixtures co-processed by fluid-bed melt granulation.

Sample	LMH (%)	Calcium Dihydrogen Phosphate Dihydrate (%)	Croscarmellose Sodium (%)	PEG 4000 (%)	P188 (%)	GPS (%)
10% PEG 4000	70	15	5	10	x	x
20% PEG 4000	60	15	5	20	x	x
10% P188	70	15	5	x	10	x
20% P188	60	15	5	x	20	x
10% GPS	70	15	5	x	x	10
20% GPS	60	15	5	x	x	20

**Table 2:** Flowability of co-processed mixtures, LMH and Ludipress^®^.

Sample	Compressibility Index	Hausner Ratio (%)	Flowability	
			According to Scale of Ph. Eur. 10.0 (Table 2.9.36.-2.)	Flow Rate Measured by Flow through an Orifice (g/s)
10% PEG 4000	10.76 ± 0.70	1.12 ± 0.01	good	10.87 ± 0.10
20% PEG 4000	8.25 ± 0.95	1.09 ± 0.01	excellent	9.59 ± 0.25
10% P188	9.14 ± 0.35	1.10 ± 0.01	excellent	10.66 ± 0.20
20% P188	6.69 ± 0.90	1.07 ± 0.01	excellent	9.40 ± 0.32
10% GPS	13.72 ± 2.24	1.16 ± 0.03	good	7.41 ± 0.09
20% GPS	8.22 ± 0.59	1.09 ± 0.01	excellent	8.82 ± 0.13
LMH	35.70 ± 1.12	1.55 ± 0.03	very poor	1.24 ± 0.13
Ludipress^®^	15.52 ± 2.66	1.18 ± 0.04	fair	7.46 ± 0.09

**Table 3:** Detachment parameters for co-processed mixtures, LMH and Ludipress^®^.

Sample	Detachment Parameters	Compression Pressure (MPa)				
		34.7	69.4	104.1	138.8	173.5
10% PEG 4000	DW (×10^−2^ J)	7.90 ± 4.43	16.06 ± 5.86 ^b^	21.58 ± 7.36 ^a,b^	36.64 ± 11.00 ^a,b^	44.13 ± 12.62 ^a,b^
	DS (MPa)	1.51 ± 0.27	2.79 ± 0.09 ^b^	3.75 ± 0.32	4.35 ± 0.76	4.68 ± 0.59
20% PEG 4000	DW (×10^−2^ J)	4.00 ± 2.05	11.52 ± 2.87 ^b^	22.09 ± 5.85 ^a,b^	25.55 ± 5.63 ^a,b^	32.13 ± 2.39 ^a,b^
	DS (MPa)	1.53 ± 0.42	3.27 ± 0.34	4.20 ± 0.39	4.08 ± 1.36	4.91 ± 0.38
10% P188	DW (×10^−2^ J)	2.23 ± 1.58 ^b^	6.12 ± 4.21 ^a,b^	13.29 ± 9.15 ^a,b^	13.73 ± 5.35 ^a,b^	15.98 ± 5.88 ^a,b^
	DS (MPa)	0.77 ± 0.12 ^a,b^	1.59 ± 0.19^b^	2.45 ± 0.22 ^a,b^	2.66 ± 0.63 ^a,b^	2.89 ± 0.53 ^a,b^
20% P188	DW (×10^−2^ J)	2.60 ± 0.95 ^b^	3.84 ± 1.22 ^a,b^	5.67 ± 2.56 ^a,b^	9.77 ± 7.25 ^a,b^	10.25 ± 6.45 ^a,b^
	DS (MPa)	1.27 ± 0.33	1.87 ± 0.50 ^b^	2.24 ± 0.70 ^a,b^	2.88 ± 0.10 ^a,b^	2.59 ± 0.67 ^a,b^
10% GPS	DW (×10^−2^ J)	1.03 ± 0.57 ^a,b^	1.72 ± 0.77 ^a,b^	2.83 ± 1.20 ^a,b^	3.23 ± 1.13 ^a,b^	2.96 ± 1.15 ^a,b^
	DS (MPa)	0.71 ± 0.10 ^a,b^	1.08 ± 0.16 ^b^	1.14 ± 0.26 ^a,b^	1.31 ± 0.46 ^a,b^	1.25 ± 0.47 ^a,b^
20% GPS	DW (×10^−2^ J)	1.06 ± 0.48 ^a,b^	0.86 ± 0.17 ^a,b^	1.06 ± 0.50 ^a,b^	1.02 ± 0.41 ^a,b^	1.21 ± 0.29 ^a,b^
	DS (MPa)	0.68 ± 0.11 ^a,b^	0.76 ± 0.06^b^	0.72 ± 0.22 ^a,b^	0.82 ± 0.22 ^a,b^	1.00 ± 0.14 ^a,b^
LMH	DW (×10^−2^ J)	4.77 ± 2.35	15.17 ± 7.76 ^b^	97.57 ± 47.92	144.55 ± 59.15	139.61 ± 84.42
	DS (MPa)	1.40 ± 0.47	1.13 ± 0.54 ^b^	3.95 ± 1.22	5.79 ± 1.65	5.16 ± 1.87
Ludipress^®^	DW (×10^−2^ J)	10.43 ± 6.85	47.74 ± 13.78	53.42 ± 14.16	77.92 ± 39.95 ^a^	111.64 ± 36.83
	DS (MPa)	1.71 ± 0.42	4.18 ± 1.05	4.57 ± 1.09	5.56 ± 2.03	8.02 ± 3.49

Statistically significant (*p* < 0.05) lower values relative to ^a^ LMH and ^b^ Ludipress^®^.

**Table 4:** Ejection parameters for co-processed mixtures, LMH and Ludipress^®^.

Sample	Ejection Parameters	Compression Pressure (MPa)				
		34.7	69.4	104.1	138.8	173.5
10% PEG 4000	EW (×10^−2^ J)	4.21 ± 0.69 ^a,b^	7.16 ± 1.43 ^a,b^	7.15 ± 0.56 ^a,b^	8.46 ± 0.68 ^a,b^	9.22 ± 0.69 ^a,b^
	ES (MPa)	0.94 ± 0.06 ^a,b^	1.68 ± 0.16 ^a,b^	1.78 ± 0.16 ^a,b^	3.18 ± 0.08 ^a,b^	4.29 ± 0.47 ^a,b^
20% PEG 4000	EW (×10^−2^ J)	2.96 ± 0.42 ^a,b^	4.35 ± 0.93 ^a,b^	5.59 ± 1.06 ^a,b^	5.97 ± 1.76 ^a,b^	6.39 ± 2.13 ^a,b^
	ES (MPa)	0.89 ± 0.18 ^a,b^	1.70 ± 0.15 ^a,b^	3.02 ± 0.64 ^a,b^	3.10 ± 0.61 ^a,b^	3.58 ± 0.94 ^a,b^
10% P188	EW (×10^−2^ J)	3.14 ± 0.84 ^a,b^	4.31 ± 0.24 ^a,b^	6.02 ± 0.77 ^a,b^	6.87 ± 0.72 ^a,b^	6.91 ± 0.45 ^a,b^
	ES (MPa)	0.52 ± 0.11 ^a,b^	1.14 ± 0.19 ^a,b^	1.76 ± 0.25 ^a,b^	1.92 ± 0.27 ^a,b^	2.06 ± 0.19 ^a,b^
20% P188	EW (×10^−2^ J)	3.09 ± 0.52 ^a,b^	3.66 ± 0.21 ^a,b^	3.61 ± 0.22 ^a,b^	3.88 ± 0.17 ^a,b^	3.96 ± 0.53 ^a,b^
	ES (MPa)	0.70 ± 0.15 ^a,b^	0.89 ± 0.17 ^a,b^	1.07 ± 0.19 ^a,b^	1.51 ± 0.36 ^a,b^	1.66 ± 0.55 ^a,b^
10% GPS	EW (×10^−2^ J)	1.48 ± 0.28 ^a,b^	2.40 ± 0.54 ^a,b^	2.50 ± 0.23 ^a,b^	2.81 ± 0.26 ^a,b^	2.93 ± 0.24 ^a,b^
	ES (MPa)	0.31 ± 0.08 ^a,b^	0.46 ± 0.02 ^a,b^	0.55 ± 0.08 ^a,b^	0.68 ± 0.14 ^a,b^	0.73 ± 0.10 ^a,b^
20% GPS	EW (×10^−2^ J)	0.79 ± 0.19 ^a,b^	1.00 ± 0.12 ^a,b^	1.22 ± 0.25 ^a,b^	1.28 ± 0.21 ^a,b^	1.34 ± 0.20 ^a,b^
	ES (MPa)	0.17±0.02^ab^	0.20 ± 0.01 ^a,b^	0.24 ± 0.03 ^a,b^	0.26 ± 0.04 ^a,b^	0.29 ± 0.04 ^a,b^
LMH	EW (×10^−2^ J)	15.10 ± 5.54	30.67 ± 4.18 ^b^	131.22 ± 23.20	142.80 ± 16.73	156.06 ± 8.13
	ES (MPa)	1.30 ± 0.31	2.66 ± 0.23 ^b^	11.84 ± 3.56	15.91 ± 4.15	18.90 ± 4.02
Ludipress^®^	EW (×10^−2^ J)	20.38 ± 11.67	120.21 ± 41.44	139.90 ± 37.30	137.85 ± 38.29	165.53 ± 77.82
	ES (MPa)	1.33 ± 0.59	7.68 ± 2.71	9.57 ± 3.56	13.82 ± 6.40	13.35 ± 4.98

Statistically significant (*p* < 0.05) lower values relative to ^a^ LMH and ^b^ Ludipress^®^.

**Table 5:** Disintegration time of compacts prepared by compression of co-processed mixtures, LMH and Ludipress^®^ at compression pressure of 173.5 MPa.

Sample	Disintegration Time (min)
10% PEG 4000	1.88
20% PEG 4000	4.07
10% P188	2.33
20% P188	5.45
10% GPS	0.57
20% GPS	10.97
LMH	1.77
Ludipress^®^	5.25

## Data Availability

The data presented in this study are available on request from the corresponding author.
